# Formulation and Biodegradation of Surface-Supported Biopolymer-Based Microgels Formed via Hard Templating onto Vaterite CaCO_3_ Crystals

**DOI:** 10.3390/ma17010103

**Published:** 2023-12-25

**Authors:** Mariam Mammen, Cain Hogg, Dominic Craske, Dmitry Volodkin

**Affiliations:** 1 Department of Chemistry and Forensics, School of Science and Technology, Nottingham Trent University, Clifton Lane, Nottingham NG11 8NS, UK; mariam.mammen2022@my.ntu.ac.uk (M.M.); cain.hogg2020@my.ntu.ac.uk (C.H.); 2 School of Science and Technology, Nottingham Trent University, Clifton Lane, Nottingham NG11 8NS, UK; dominic.craske@ntu.ac.uk

**Keywords:** multilayer, layer-by-layer, calcium carbonate, polyelectrolyte, enzyme

## Abstract

In recent decades, there has been increased attention to the role of layer-by-layer assembled bio-polymer 3D structures (capsules, beads, and microgels) for biomedical applications. Such free-standing multilayer structures are formed via hard templating onto sacrificial cores such as vaterite CaCO_3_ crystals. Immobilization of these structures onto solid surfaces (e.g., implants and catheters) opens the way for the formulation of advanced bio-coating with a patterned surface. However, the immobilization step is challenging. Multiple approaches based mainly on covalent binding have been developed to localize these multilayer 3D structures at the surface. This work reports a novel strategy to formulate multilayer surface-supported microgels (ss-MG) directly on the surface via hard templating onto ss-CaCO_3_ pre-grown onto the surface via the direct mixing of Na_2_CO_3_ and CaCl_2_ precursor solutions. ss-MGs were fabricated using biopolymers: polylysine (PLL) as polycation and three polyanions—hyaluronic acid (HA), heparin sulfate (HS), and alginate (ALG). ss-MG biodegradation was examined by employing the enzyme trypsin. Our studies indicate that the adhesion of the ss-MG to the surface and its formation yield directly correlate with the mobility of biopolymers in the ss-MG, which decreases in the sequence of ALG > HA > HS-based ss-MGs. The adhesion of HS-based ss-MGs is only possible via heating during their formation. Dextran-loading increases ss-MG formation yield while reducing ss-MG shrinking. ss-MGs with higher polymer mobility possess slower biodegradation rates, which is likely due to diffusion limitations for the enzyme in more compact annealed ss-MGs. These findings provide valuable insights into the mechanisms underlying the formation and biodegradation of surface-supported biopolymer structures.

## 1. Introduction

Over the recent decades, there have been extensive studies in drug formulation, especially regarding targeted drug delivery while maintaining therapeutic effects. Currently, biopolymer-based layer-by-layer (LbL) assembled capsules (usually hollow structures) or microgels (MG) filled with biopolymers are being used for drug encapsulation and controlled drug release by stimuli [1]. Specifically, many research efforts have been directed toward the development of smart delivery systems capable of consistent drug release patterns in response to precise stimuli [2]. As for the advantage of using MGs, they have the potential to combine a variety of materials with different properties and build biological functionality in one unique delivery system [3]. The interest in LbL assembly arises not only from the ability to manipulate surface characteristics but also due to its potential in the synthesis of polymeric nano- or micro-materials [4]. These MGs are generated through the process of adsorbing oppositely charged polyelectrolytes onto a sacrificial template, facilitated by electrostatic interactions, followed by decomposition of the sacrificial template (so-called hard templating) and are extensively used for drug delivery and other applications [5,6,7,8]. In this context, one of the most popular sacrificial templates/cores comprises mesoporous calcium carbonate vaterite crystals fabricated by mixing two salt solutions, Na_2_CO_3_ and CaCl_2_, which are chosen for their attributes of biocompatibility, non-toxic, cost-effective, easy formulation, and scalability [5,9,10,11,12].

Polymer-based MG formation was introduced a decade ago with the formation of matrix-type capsules. MGs were formed by the LbL deposition of oppositely charged polymers onto a sacrificial vaterite CaCO_3_, followed by core dissolution by EDTA or at a pH below neutral [13,14]. As a result, a polyelectrolyte network is established on the surface and within the porous core through the diffusion of polyelectrolytes, thereby giving rise to what is referred to as matrix-type capsules formed after the core decomposition [15,16,17]. In general, vaterite-based hard templating has attracted significant scientific attention in the past due to the biocompatible conditions used to decompose the templates, offering an option to work with fragile (bio)molecules to form micro-sized particles loaded with sensitive proteins and enzymes as well as other biologically active small and large molecules of different natures [18,19,20,21,22,23,24,25]. Moreover, the internal structure of the vaterite crystals allows for the forming of an inverted replica of the solid crystals made of soft polymers [26] or hard nanoparticles [27,28,29,30,31,32].

Furthermore, the stability of MGs is intricately linked to the characteristics of polyelectrolyte complexes, necessitating careful consideration during their fabrication for drug delivery and various other biomedical applications [1]. Several studies have highlighted the utmost significance of polyelectrolyte properties in preparing multilayer MGs. The choice of a polyelectrolyte is intricately linked to its intended application and inherent properties [33]. Recent studies have reported tailor-made MGs and capsules formed from various polyelectrolytes—synthetic and biologically derived or biologically relevant—as well as their combinations. These include the pairs of hyaluronic acid (HA)/poly(allylamine) (PAH), HA/poly(lysine) (PLL) [34], poly-l-arginine (pARG)/dextran sulfate (DEXS), poly (hydroxypropylmethacrylamide dimethylaminoethyl) (p(HPMA-DMAE)/polystyrene sulfonate (PSS) [35], PAH/PSS [36], and PSS/poly (diallyldimethylammonium chloride) PDAD) [15].

Typically, the formed multilayer MGs and capsules are free-standing structures. A distinctive aspect of recently reported biopolymer-based MGs is their ability to adhere to the surface during the core removal process [1]. The mechanism of the adhesion has been explained in the context of polymer annealing [1,37]. In order to reveal the MG formation mechanism and their adhesion to the surface, Campbell et al. conducted a detailed comparative study on 16 combinations of polyelectrolyte pairs of biopolymers fabricated onto vaterite crystals [1]. The formulation of surface-supported (ss) microgels (ss-MGs) provides significant advantages for patterning biologically relevant surfaces, e.g., coatings of implants with biopolymers having a high level of control over the coating degree and the composition of the coatings. This is indispensable for better integration of the implants via tuning the implant–tissue interface properties and composition. Such coatings can serve as advanced biodegradable tailor-made scaffolds for successful implantation. For such coating formulations, the simplicity, tuning of the coating structure, and the enzyme -mediated biodegradation play a pivotal role.

Considering the intricate mechanism of enzymatic degradation, the concept of drug delivery through multilayer assembled biopolymer-based structures emerges as a highly promising strategy. This approach capitalizes on the process of layer breakdown, resulting in a more localized and controlled release of the drug payload [38]. The underlying principle of enzyme degradation harnesses the presence of enzymes within the body’s physiological environment in specific anatomical regions [39]. These enzymes interact with the multilayers, instigating their degradation and the subsequently controlled release of the encapsulated drug. The appeal of enzyme-triggered degradation as a foundation for drug delivery is gaining considerable attention in the pharmaceutical sector. The interest arises from the fact that the enzymes are not uniformly distributed throughout the body but rather tend to cluster within distinct anatomical regions. This spatial concentration aligns with the localized nature of enzymatic activity. Consequently, the approach enhances the precision, specificity, and overall drug release, which is essential in the principle of drug delivery technology. In comparison to externally triggered methods for drug release, biologically responsive approaches hold greater appeal within drug delivery. In this context, a biologically responsive capsule was highlighted, utilizing biopolymer protamine sulfate (PRM) and heparin (HEP) by coating them onto sacrificial CaCO_3_ microparticles. These PRM/HEP microcapsules are designed to react in the presence of the biological enzyme trypsin, leading to the degradation of the protamine layer and resulting in the release of encapsulated dextran. This design offers the distinct advantage of employing a highly localized trigger, as enzymes are often overexpressed within a cancerous environment. Employing an enzymatically triggered degradation system proves to be more efficient than a sustained release system when aiming to effectively deliver drugs to cancerous tumors [40]. 

Prior research, as per the discussion above, has utilized the concept of MGs supported on the surface and the importance of biodegradation studies of multilayer biopolymer-based structures. As described above, so far, SS-MGs have only been reported to be formed when the multilayer coating steps have taken place for free-standing vaterite crystals collected using centrifugation [1]. This study aims to develop an approach for the straightforward formulation of MGs performed completely on the surface to form so-called ss-MGs via ss-crystals of vaterite CaCO_3_ grown directly on the surface. Therefore, specific research objectives were set: (i). To design biopolymer-based ss-MGs fully formulated on a surface employing PLL as a polycation with three polyanions (HA, ALG, and HS) (ii). To characterize the ss-MG physical–chemical properties, including formation yield, adhesion, morphology, and sizes, using fluorescence/optical, electron-based, and atomic force microscopies (iii). To assess ss-MG resistance against freezing for ss-MG storage (iv). To investigate ss-MG biodegradation and the drug release mechanism using dextran as a model macromolecular drug and trypsin as a proteinase enzyme that is able to cleave/degrade biopolymer backbones. 

The proposed approach would eliminate the need for multiple centrifugation steps required to deposit multilayers (standard procedure to separate coated particles from non-adsorbed polymers). It would make the process of MG formulation easy and attractive for industrial applications via the simple exposure of polymer solutions to the surface where the LbL deposition of ss vaterite crystals takes place. Here, we conduct a comparative analysis of the properties of MGs made of various biopolymers: polyanions hyaluronic acid (HA), alginic acid (ALG), and heparin sulfate (HS) combined with polycation polylysine (PLL). Additionally, ss-MG biodegradation in the presence of an enzyme has been examined using fluorescence microscopy imaging. 

## 2. Experimental Section

### 2.1. Materials

Calcium chloride dihydrate (Acros Organics, Loughborough, UK, catalog number 10158280), Sodium carbonate (Acros Organics, Loughborough, UK, catalog number 10577182), Sodium chloride (Fisher Scientific, Loughborough, UK, catalog number, 10316943), EDTA (ethylenediaminetetraacetic acid), (Fisher Scientific, Loughborough, UK, catalog number 10335460), Tris 10×—250 mM Tris containing 27 mM KCl, 1370 mM NaCl, pH 7.4 (Alfa Aesar, Ward Hill, MA, USA, catalog number J60764), Hyaluronic acid (HA) 250 kDa (Creative PEGWorks, Chapel Hill, NC, USA, catalog number HA-103). Alginic acid (ALG) (catalog number A-0682), Heparin sulfate (HS) (catalog number H5515), Poly-L-lysine hydrobromide (PLL) 15–30 kDa (catalog number P7890), Ethylenediaminetetraacetic acid (EDTA) (catalog number E6758), 0.25% Trypsin-EDTA solution (catalog number T4049–100 ML), Fluorescein isothiocyanate–dextran of MW 2000 kDa (DEX-FITC) (catalog number FD2000S) were from Merck, Gillingham, UK.

### 2.2. Synthesis of ss-CaCO_3_ Vaterite Crystals

#### 2.2.1. Two-Step Synthesis

The two-step synthesis is based on formation of vaterite nuclei in separate wells followed by replacing the solution into/onto any other surface of interest where crystals will further grow. In 24-well cell culture plate, 200 µL of 150 mM CaCl_2_, 200 µL of 150 mM Tris 6×, and 200 µL of pure water were added with a magnetic stirrer. The solution was agitated on the magnetic stirrer for around 5 min at 100 rpm. Afterward, without ceasing the stirring, 600 µL of 50 mM Na_2_CO_3_ was added to the solution of CaCl_2_, and agitation completely ceased after 15 s. After the addition of Na_2_CO_3_, the solution becomes turbid in appearance, denoting the formation of CaCO_3_. Immediately after, 50 µL of the turbid solution was placed in the 96-well cell culture plate and performed in two more wells. The samples were left undisturbed for 40 min, allowing the vaterite CaCO_3_ particles to adhere to the surface.

#### 2.2.2. One-Step Synthesis

The one-step synthesis is simpler compared to the two-step synthesis (above) since it allows for direct growth of crystals on the surface present in contact with the mixed salt precursor solutions. It has limitations of using only that surface where salts can be properly mixed to form nuclei of vaterites followed by crystal growth. In this case, cell culture plates have been used, and crystal growth occurred directly in the wells of the plates. The crystallization process was initiated by rapid mixing of pre-filtered (through 0.22 µm syringe filter) CaCl_2_ and Na_2_CO_3_ water solutions at room temperature in 96-well plate under shaking. For this, the following solutions (A–C) were placed into a well: A—0.02 mL of 150 mM CaCl_2_; B—0.02 mL of 150 mM TRIS (containing 16 mM KCl, 820 mM NaCl), pH 7.4 (TRIS 6×); C—0.02 mL water or 2 mg/mL dextran^FITC^ in water solution. The final solution has been shaken at 700 rpm. Then, solution D (0.06 mL of 50 mM Na_2_CO_3_) was added, followed by 60 s of further shaking. When shaking stopped, crystals were left untouched to grow for 30 min.

### 2.3. Fabrication of ss-MGs

After 40 min of ss-CaCO_3_ settling onto the surface, they were washed with 50 µL of 0.2× Tris buffer three times. The 0.2× Tris buffer solution was prepared from 10× concentration solution, and pH was adjusted to obtain 5 mM Tris, 0.5 mM KCl, 10 mM CaCl_2_, and pH 8.9. For ALG-based ss-MGs, 37 mM NaCl was used in the buffer instead of 10 mM CaCl_2_ to avoid gelation of ALG in bulk solution. Then 50 µL of 0.2× Tris buffer was added, followed by addition of 50 µL of 0.5 mg/mL of polyanion in 0.2× Tris buffer. After addition of the solution, the well plate was shaken for 700 rpm for 14 min. Once the shaking was performed, the supernatant was removed, and the well was washed with 150 µL of 0.2× Tris and then shaken for 5 min at 700 rpm. The washing solution was removed, and the next deposition layer started with addition of 50 µL of 0.2× TRIS followed by 50 µL of PLL as polycation in 0.2× Tris buffer. The deposition of polyanion and PLL was repeated until 5 layers were deposited, giving the structure (polyanion/PLL)_2_/polyanion. Once the 5 layers were made, 50 µL of 0.2× Tris buffer was added along with slow drop-wise addition of 100 µL of 50 mM EDTA. This resulted in dissolution of carbonate core and formation of ss-MGs. In case of HS-based MGs, the formed MGs were heated to 70 °C for 30 min, which resulted in their adhesion to surface; otherwise (without heating step), the MGs were removed from surface after their formation via gentle washing.

### 2.4. Biodegradation of MGs

The batch of coated ss-CaCO_3_ was prepared and stored in the fridge. They were stored overnight, and the CaCO_3_ cores were dissolved with EDTA from the microgels the next day. These microgels were evaluated for biodegradation as a part of their drug-releasing capability. The multilayer polymer ss-MGs were degraded with 100 µL of 0.25% Trypsin-EDTA, pH 7.0–7.6, as per product description. The images of the same position and fluorescence conditions were taken for different time intervals. The reduction in the fluorescence during the biodegradation is associated with release of DEX-FITC from the ss-MGs. The mechanism of release is discussed below and is based on cleavage of PLL by protease trypsin, as PLL is a polyaminoacid. 

### 2.5. Optical and Fluorescence Microscopy

All the images of the microgels were taken with the optical microscope EVOS from Fisher Scientific, UK. They were characterized with the help of EVOS color imaging system that provided transmittance and fluorescence images of the microgels. Further analysis of the microgel properties and the evaluation of trypsin’s impact on them were performed using the ImageJ 1.44c software. Properties, including average diameter, formation yield, shrinking coefficient, and adhesiveness, were quantified with this software. The calculation of these properties was performed for these microgels. The average diameter, calculated for 30 microgels, was calculated by the difference in diameter between crystals and microgels.

$$Diameter=2\sqrt{\frac{Area}{\pi }},$$

Additionally, the data for the average diameter of the crystals and microgels were used to estimate the shrinkage coefficient of the microgels. Previous studies have shown shrinkage of microgels after dissolution of CaCO_3_ core depending on the polymers used. As a result, the unitless shrinking coefficient was considered and calculated in this study as per the below.

$$Shrinking\;Coefficient=\frac{Average\;diamter\;of\;crystals\;(µm)}{Average\;diamter\;of\;microgels(µm)}$$

Furthermore, the number of microgels formed from the crystals is used to calculate the formation yield in percentage. The formula for determining the formation yield of the microgels is as follows:

$$Formation\;Yield\;\left(\%\right)=\frac{Number\;of\;microgels}{Number\;of\;crystals}$$

Given that these microgels are supported on the surface, evaluating their adhesiveness is important. The adhesiveness is quantified as a percentage, indicating the microgel’s resistance to being washed away with buffer solution during the ss-MG formation. The calculation for adhesiveness is carried out using the following formula:

$$Adhesiveness\;\left(\%\right)=\frac{Number\;of\;microgels\;after\;washing}{Number\;of\;microgels\;before\;washing}$$

### 2.6. Scanning Electron Microscopy (SEM)

Morphology of ss-MGs has been analyzed using a JEOL JSM-7100f SEM, Tokyo, Japan. Samples were prepared on chamber slides containing 10-cell culture plate and were subsequently left to air-dry. To enhance conductivity, a 5 nm thick gold coating was applied to the specimens via sputter coating (Quorum Q150R ES, Lambda Photometrics Ltd., Harpenden, UK). Following this, the samples were mounted onto the sample holder using copper tape and double-sided carbon tape below the slide. Images of the samples were collected in secondary electron imaging mode at an accelerating voltage of 5 kV.

### 2.7. Atomic Force Microscopy (AFM)

A Bruker Dimension Icon scanning probe microscope (SPM) was utilized to investigate the sample surface topography. Samples were prepared on a glass slide and allowed to air-dry. AFM in tapping mode was carried out using a ScanAsyst-Air (supplied by Bruker, Coventry, UK) probe. Data visualization and analysis were performed using Gwyddion, version 2.64.

## 3. Results and Discussion

### 3.1. Synthesis of ss-CaCO_3_ Vaterite Crystals and ss-MGs

**Figure 1:**
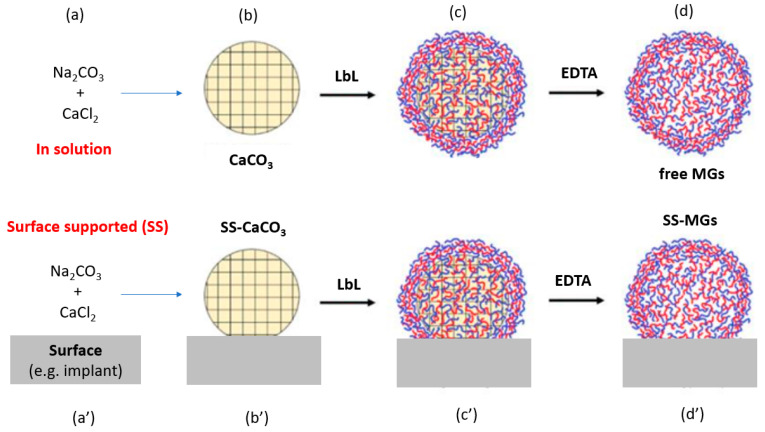
Schematic representation of formation of free-standing MGs (**a**–**d**) and ss-MGs (**a’**–**d’**) via vaterite-based hard templating in solution and in presence of solid surface, respectively. The “LbL” stands for “layer-by-layer” which is the deposition process to form multilayers into pores of CaCO_3_ crystals.

The ss-CaCO_3_ crystals have been prepared via a newly developed approach where the crystals have been grown onto a solid surface in the wells of cell culture plates (Figure 1). This is an alternative method to form vaterite crystals not in bulk but to grow them on a solid surface when precursor salts are mixed in the presence of a surface directly (one-step synthesis) or in separate wells followed by replacement to another surface (two-step synthesis). Further, the two-step synthesis has been used unless specified. For this, the preformed nuclei of vaterites (heterogeneous nucleation) were first produced in 24-well cell culture plates via the mixing of CaCl_2_ and Na_2_CO_3_ through stirring. This was followed by the quick replacement of the solution with nuclei to another surface (96-well cell culture plates), where the final crystals have been grown on the surface, so they are finally bound to the surface (Figure 1). The vaterite crystals were prepared according to the protocol to form the optimal size and size distribution essential for analysis. The growth of vaterite crystals is performed at supersaturated conditions (the concentration of used precursor salts is orders of magnitude more than the concentration of calcium or carbonate ions in the solution after the crystal’s growth), which means the yield of the crystal formation can be assumed to be nearly 100%.

**Figure 2:**
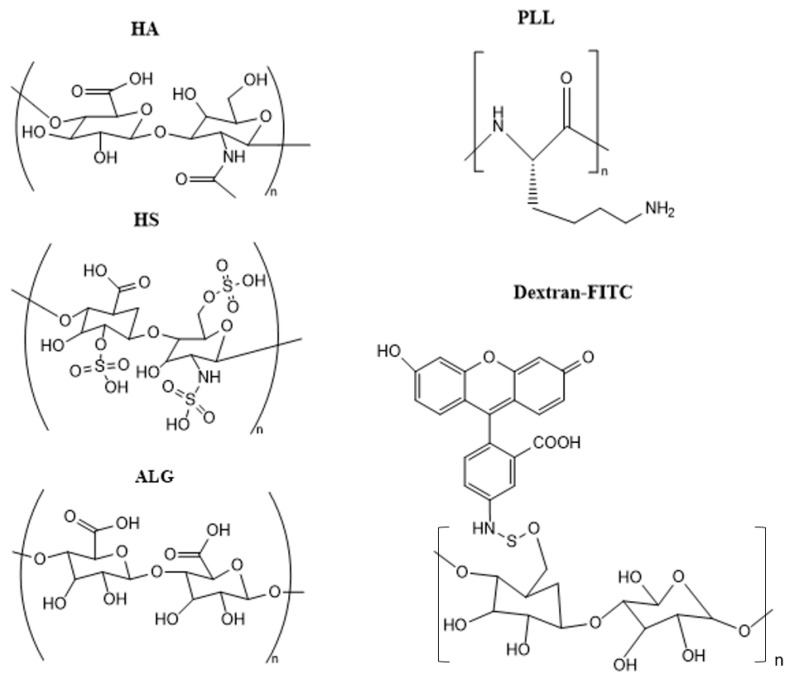
Molecular structures of biopolymers used to formulate MGs (polyanions hyaluronic acid (HA), heparin sulfate (HS), alginic acid (ALG) and polycation poly-L-Lysine (PLL) as well as Dextran-FITC used as model macromolecule to encapsule into MGs.

The MGs have been formed by the multilayer coating of the ss-CaCO_3_ crystals through the simple addition of biopolymers and rinsing buffer solutions into the wells of 96-well cell culture plates. The deposition of biopolymers has been performed in a tris-buffer solution, pH 8.9, to ensure that pH variation cannot affect the properties of the microgels as the polymer charge can depend on the pH, and the presence of CaCO_3_ will make the pH rise to ca 10–11 due to hydrolysis. PLL can have less charge at pH 8.9 as it is close to the isoelectric point of the primary amino group; however, the formation of the ss-MGs is good evidence of a multilayer formation; also, a lower charge of PLL would make it less coiled (reduced intrapolymer repulsive interactions), enhancing diffusion inside the pores of the vaterite crystals. Hyaluronic acid (HA), alginic acid (ALG), and heparin sulfate (HS) were used as polyanions, and polylysine (PLL) was used as polycation to form three pairs of five-layer ss-MGs. Therefore, the final structure of ss-MGs can be expressed as (HA/PLL)_2_/HA, (ALG/PLL)_2_/HA, and (HS/PLL)_2_/HA. Figure 2 illustrates the structure of the biopolymers used for the fabrication of the ss-MGs, along with fluorescently labeled dextran used as a model macromolecular drug (DEX-FITC). Polyanions have been chosen as polysaccharides (they have the same structural similarity as glucose-based disaccharides), and they differ by chemical group; for example, there is a carboxylic group (for HA and ALG) and a sulfonate group for HS. We believe that EDTA cannot be left in ss-MGs in considerable amounts as interpolymer interactions are stronger due to a stronger cooperative character than the interaction of EDTA with polymers, and EDTA will be excluded from the annealing process taking place in this study. However, some residual amounts of EDTA may be present, and this should be considered in the case of application of the fabricated ss-MGs where there are restrictions for the use of EDTA.

### 3.2. Properties of ss-CaCO_3_ and ss-MGs

**Figure 3:**
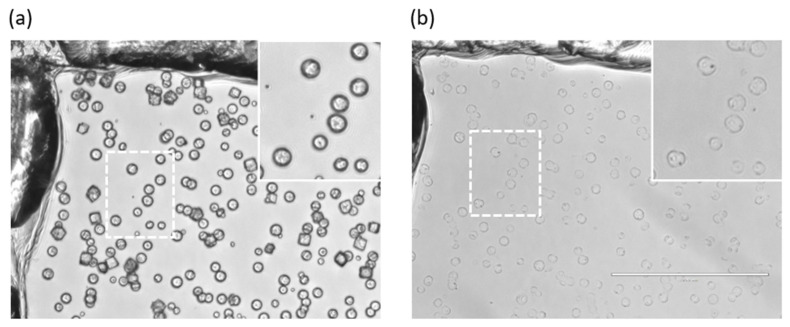
Optical microscope images of (**a**) ss-CaCO_3_ crystals synthesized and (**b**) ALG-based ss-MGs. The scratch in the upper left corner of the images is made using a needle and is only used as a marker since it is easy to navigate. Scale bar is 200 µm.

Figure 3 demonstrates the appearance and shape of vaterite crystals and ALG-based ss-MGs from the transmittance images. One can clearly see spherical particles of ss-CaCO_3_ and many more transparent ss-MGs since the carbonate core is dissolved for ss-MGs (calcium carbonate has a refractive index of ca 1.5–1.6, which is much more than that of water, which is 1.33). ss-CaCO_3_ can host DEX-FITC, which is distributed rather homogeneously in the crystals (Figure S1). Transmittance images of HA- and HS-based ss-MGs are presented in Figures S2–S4.

For the fabrication of ALG polymer microgels, 37 mM NaCl is used instead of 10 mM CaCl_2_ in the buffer solution, as the presence of CaCl_2_ results in the gelation of ALG in a bulk solution. All types of MGs formed have been surface-supported structures. HA- and ALG-based ss-MGs adhered to the surface very well after their formation. However, HS-based ones did not adhere to the surface after their formation (they were free-standing). As for the fabrication of HS-based MGs, in order to facilitate their adherence to the surface, a subsequent step involves subjecting the microgels to a temperature of 70 °C for a duration of 30 min, which resulted in their strong adhesion to the surface.

Table 1 below summarizes the average diameters of the ss-CaCO_3_ and ss-MGs templated on them. This includes bare crystals and ss-MGs as well as DEX-FITC-loaded ones. 

As one can see from Table 1, the formed ss-MGs are typically smaller than the original size of the templates (ss-CaCO_3_ crystals), and the presence of DEX-FITC influences the sizes of the formed ss-MGs. The size of DEX-FITC-laden ss-CaCO_3_ crystals is smaller than that of the unloaded crystals. This may be explained by the inducing of nucleation of vaterite growth in the presence of DEX-FITC; where the more nuclei that are formed, the smaller the average size of the crystals as each nucleus grows into a whole crystal. The interaction of DEX-FITC with polymers n ss/MGs will be discussed below in view of polymer interactions in ss-MGs.

### 3.3. Relation of Interpolymer Interactions in ss-MGs and Their Properties

#### 3.3.1. Dynamics of Polymer Chains and Charge Compensation in Multilayers 

**Figure 4:**
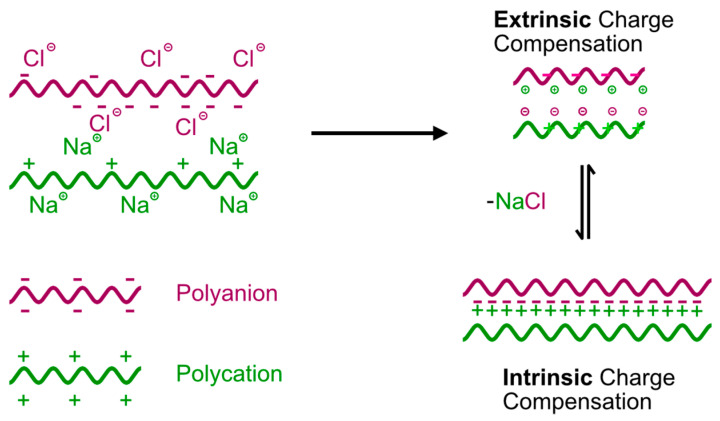
Schematics of interpolymer complex formation in the presence of salt (NaCl) that resulted in the equilibrium between intrinsically and extrinsically compensated polymer complexes.

The relationship between the ss-MG biopolymer composition and their properties is a crucial aspect associated with the biopolymer multilayer annealing effect. As for the concept of annealing, within a multilayer system, preserving a balance of net charges entails the establishment of reversible changes in two distinct forms of polymer complexes, known as extrinsically and intrinsically compensated polymer complexes (Figure 4). Schematics of the equilibrium between intrinsically and extrinsically compensated polymer complexes are shown in Figure 4 below. This equilibrium is crucial for preserving a net charge balance in multilayers. Extrinsic compensation of charges manifests polymer charges interacting with counterions from added salt, while intrinsic compensation unfolds when permanent charges on polymer backbones engage with that on another polymer molecule (ion pairs formation). We assume that this intricate interplay of oppositely charged polyelectrolytes results in a robust bonding of multilayers to the surface due to increased hydrophobicity in multilayers and water being released out of the multilayers. This phenomenon is because the multilayers undergo so-called annealing (closure of defects due to enhanced local chain dynamics and equilibrium between intrinsically and extrinsically compensated polymer complexes). The strength of this effect is associated with the specific chemistry of the polymer employed and the ionic strength in the solution, denoting the salt concentration [37]. If there are any other processes related to the compartmentation of the polymer network, e.g., due to the relaxation of the polymer structure after the removal of the vaterite cores, they are all assumed to contribute to the annealing process discussed here.

The equilibrium between intrinsically and extrinsically compensated polymer complexes plays a pivotal role in the properties of multilayers. It is affected by the external salt concentration added, but at a constant salt concentration, it will be defined by the strength of interpolymer interactions. A strong interpolymer interaction results in a “frozen” polymer complex where there is a low polymer chain mobility (dynamics) and, as a result, the annealing is restricted, so defects in the multilayer structure due to coiled polymer conformation do not change over time. Ss-MGs from such polymers would not shrink during their formation as the annealing is a reason for the shrinkage when the closure of defects results in a reduction in the total volume of the ss-MGs. The multilayers where polymer chain dynamics are enhanced (i.e., with weak interpolymer interactions) can possess significant shrinkage due to defect closures via the annealing process.

#### 3.3.2. Main Properties of ss-MGs

For three pairs of polymers used in this study, HA/PLL ss-MGs shrink very strongly with a shrinkage coefficient of ca 3–3.5, whereas ALG/PLL ss-MGs do not shrink at all. HS/PLL ss-MGs shrink about two times compared to their original diameter. This is associated with heating of the HS/PLL ss-MGs during their formation; without heating, they do not shrink but also do not adhere to the surface at all. Therefore, the heating of HS/PLL ss-MGs results in an enhanced polymer dynamic that promotes both better adhesion and stronger shrinkage as a result of the temperature-mediated annealing process. Interestingly, HA and ALG have very similar chemical structures with two carboxylic groups on polymer disaccharide units (Figure 2). However, the ss-MGs formed using HA and ALG either shrink very strongly or do not shrink at all, respectively. This can be explained by additional binding in the ALG/PLL multilayers due to the physical crosslinking of ALG polymer molecules between each other via Ca^2+^ released during MG formation (dissolution of CaCO_3_). Such Ca^2+^-induced stabilization has been reported for ALG-based pure and multilayer-formed scaffolds and is caused by the alginate gelation inside the polymer network [41,42].

**Figure 5:**
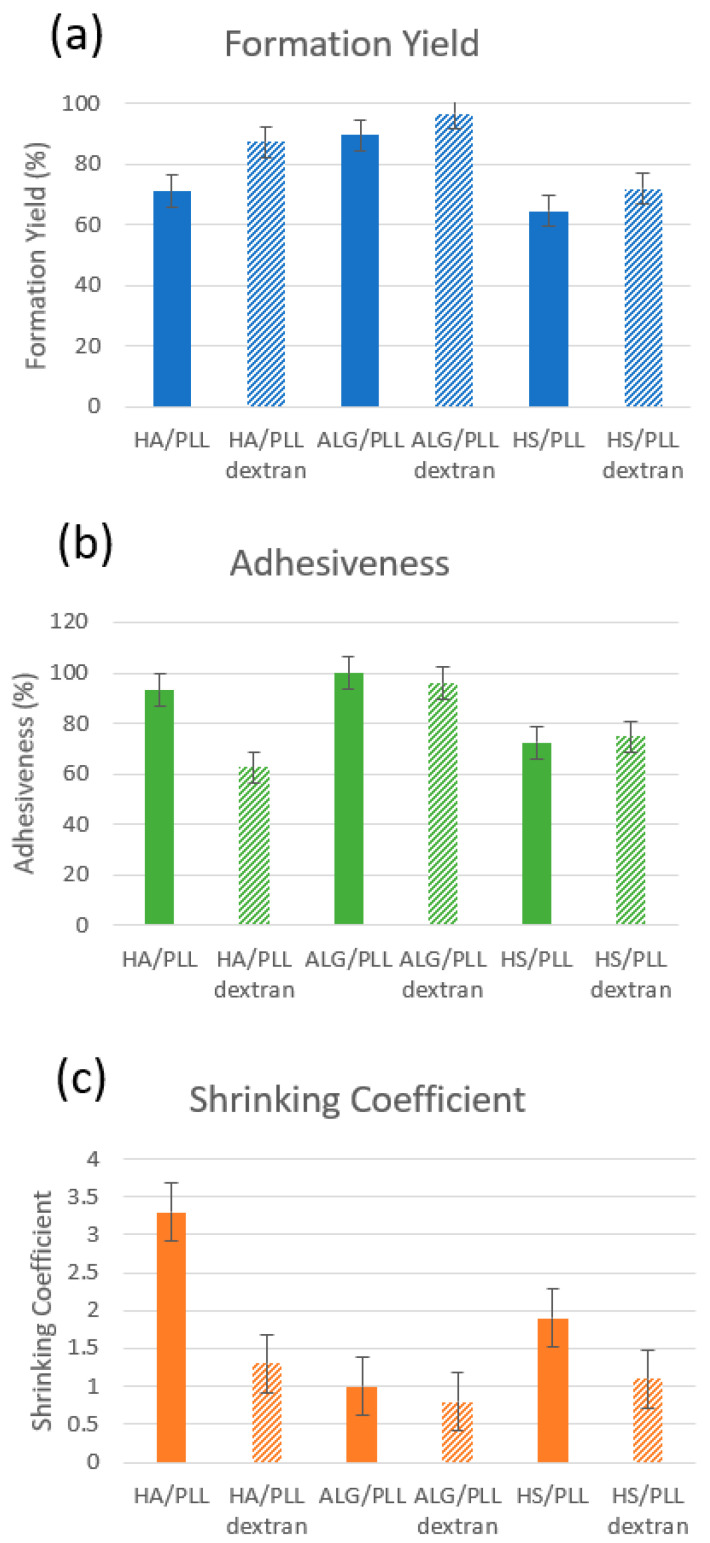
Main characteristics—formation yield (**a**), adhesiveness (**b**), shrinking coefficient (**c**)—of ss-MGs (unloaded and loaded with DEX-FITC as indicated, for instance, as HA/PLL and HA/PLL dextran in the figure) formulated in this study.

Figure 5 shows the main characteristics (formation yield, adhesiveness, shrinking coefficient) of the synthesized ss-MGs. In the context of the formation of the ss-MGs, both the formation yield and adhesion show a consistent reduction, with ALG-based ss-MGs demonstrating the highest formation yield and adhesion. This phenomenon finds its explanation in the direct connection between a successful ss-MG formation—indicated by a higher formation yield—and the effective binding of ss-MGs to the surface, exemplified by enhanced adhesion. 

The loading of dextran into ss-MGs has a notable effect on both their formation and shrinking behaviors. The incorporation of dextran increases the formation yield of MGs, likely due to the formation of smaller crystals facilitated by dextran’s presence (Table 1). Concurrently, the presence of loaded dextran impedes the shrinking of the ss-MGs (Figure 5c). This hindrance can be attributed to the physical presence of neutrally charged dextran molecules, which counteracts the shrinkage tendency and contribute to the observed reduction in shrinking behavior.

The assumption made above of the effect of the biopolymer chain dynamics on the properties of the formulated ss-MGs is well-supported by observation of the stability of ss-MG against freezing. For this test, the coated crystals were frozen at −20 °C, stored overnight, and thawed again to room temperature, followed by the addition of EDTA and formulation of ss-MGs. The fluorescence profile (distribution of biopolymer molecules across the ss-CaCO_3_) has been examined before and after the freezing procedure. Interestingly, only ALG-based ss-MGs demonstrated no changes in the biopolymer distribution against a freezing–thawing procedure. HA- and HS-based ss-MGs show a tendency towards more even polymer distribution in the coated crystals after the freezing–thawing procedure that is highly likely caused by the strong stabilization of multilayers with the additional gelation of ALG molecules as described above (Figures S5–S7).

In conclusion, it should be emphasized that the results described above point to the fact that the polymer dynamics in multilayers play a crucial role in the physical–chemical properties of the formed MGs. Polymer annealing is a process happening in complex polymer networks due to the rearrangement of the multilayer structure to thermodynamically more stable intrinsically compensated polymer complexes. This, however, is a kinetically restricted process that takes longer for multilayers with low dynamics (strong interpolymer interactions) and quicker for those with high polymer dynamics (weaker interpolymer interactions). Not only does this involve the chemistry/nature of the biopolymers, but environmental conditions (e.g., ionic strength, pH, temperature) will also contribute to the speed of the annealing process and to the mechanical properties of the ss-MGs, as this can be crucial for tissue engineering applications. The variation of these parameters can be key to designing MGs of required properties; this question will be addressed in our future work.

### 3.4. Biodegradation of ss-MGs with Trypsin

#### 3.4.1. Fluorescence Imaging

**Figure 6:**
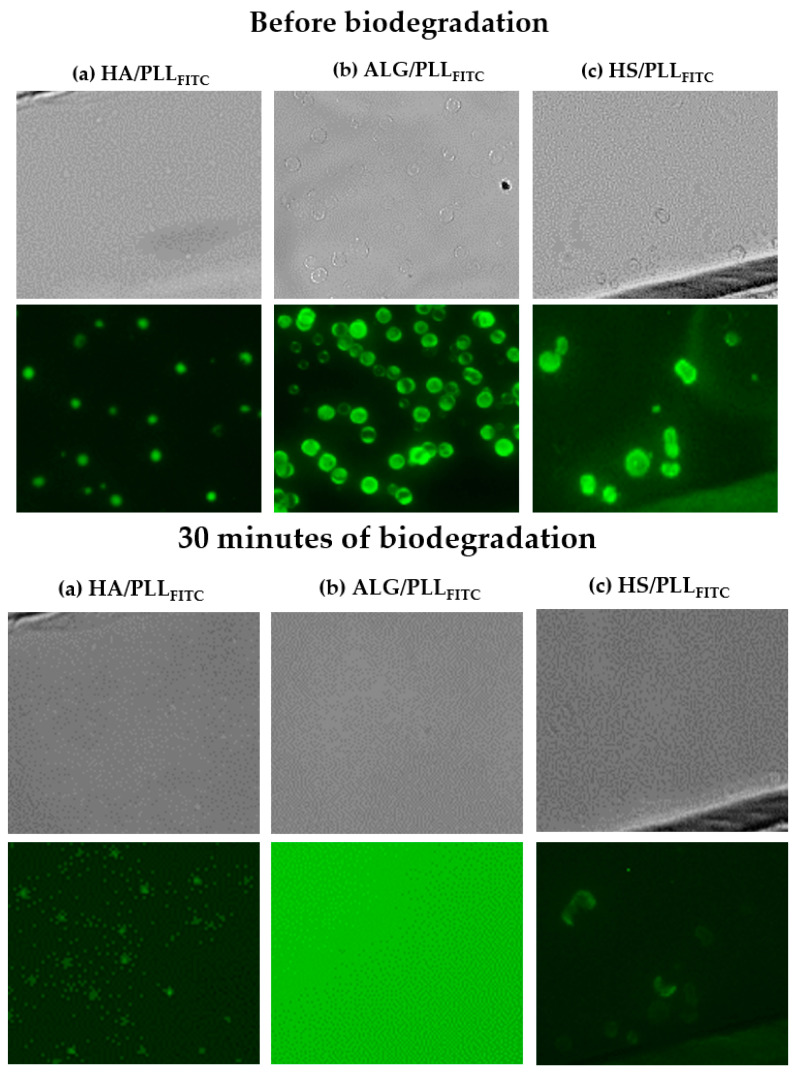
Transmittance and fluorescent images of the ss-MGs before (upper images) and after (underneath images) incubation with trypsin for 30 min. (**a**) HA/PLL_FITC_ (**b**) ALG/PLL_FITC_ (**c**) HS/PLL_FITC_ based ss-MGs. Brightness and sharpness are edited for transmitted images.

All three types of ss-MGs have been examined for their stability against biodegradation in the presence of trypsin. The ss-MGs have been prepared using fluorescently labeled PLL, i.e., PLL_FITC_. Figure 6 demonstrates how the fluorescence signal from the ss-MGs is changed after 30 min of biodegradation. The transmittance images also demonstrate that the microgels disappeared (only HA/PLL ones can be recognized) after biodegradation. Interestingly, the fluorescence is reduced for HA- and HS-based ss-MGs but significantly increased for ALG-based ones. We believe that this can be explained by the self-quenching effect. The self-quenching phenomenon [43,44,45] arises due to the presence of a high concentration of FITC fluorophores (from PLL_FITC_) inside the ALG-based ss-MGs that may be caused by the higher content of PLL in ALG-based ss-MGs caused by compact ALG gelation in the microgel polymer network, as discussed above. The ALG-based ss-MGs are less transparent compared to other types of ss-MGs produced in this work, which proves that they have a higher polymer content. PLL release at degradation results in a sudden and intense emission of light as the MGs degrade, subsequently releasing PLL_FITC_ into the surrounding environment that stops the self-quenching. This effect manifests as a rapid and pronounced flash of fluorescence (Figure 6b, after degradation).

#### 3.4.2. Biodegradation Kinetics and Mechanism

**Figure 7:**
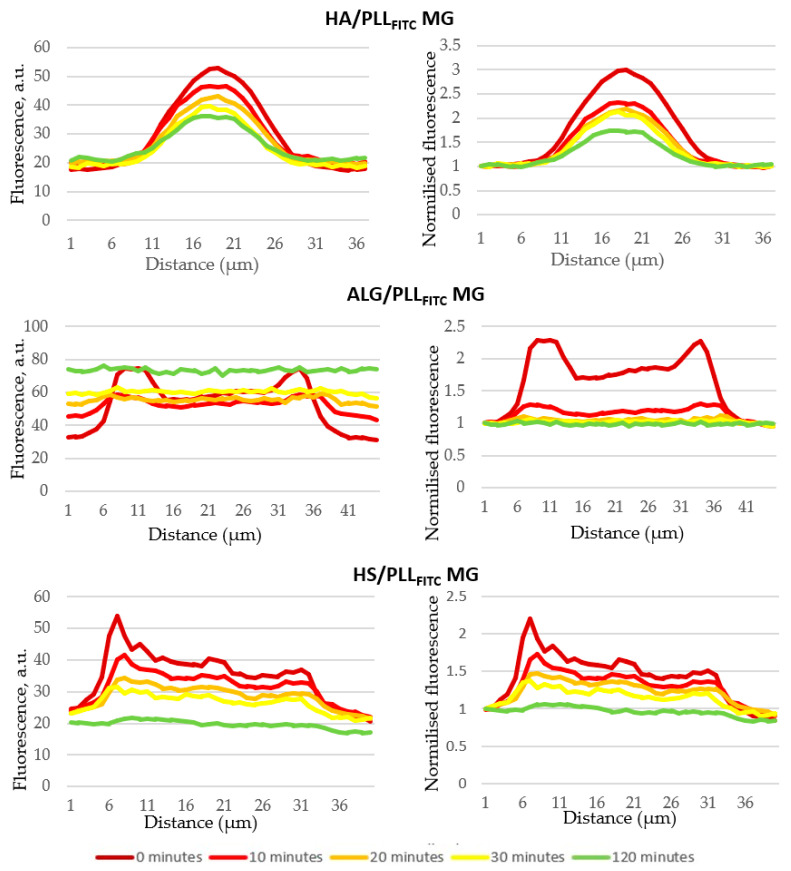
Fluorescence profile of ss-MGs at different intervals of degradation time. Normalized profiles (right-hand side, normalized to initial value of fluorescence signal) allow us to compare them between each other.

Further, the biodegradation kinetics has been assessed to better understand the influence of polymer annealing and other ss-MG properties on the capacity of trypsin to degrade the ss-MGs. Analysis of fluorescence profiles for ss-MGs during the degradation has revealed that the ss-MGs are degraded from all sites evenly as the shape of the profiles is kept at intermediate steps of degradation (Figure 7, profiles on the right-hand side). The shapes of the fluorescence profiles for all three types of ss-MGs are different, showing a cupola-like profile for HA-based ss-MGs and enriched distribution of biopolymers in the edges of the ALG- and HS-based ss-MGs. We believe that this is defined by the strength of interpolymer interactions, as discussed above. High polymer dynamics in HA-based ss-MGs make biopolymer distribution rather homogeneous due to the diffusion of polymers inside the internal volume of the ss-CaCO_3_ crystals during the multilayer deposition process. For ALG-based ss-MGs, the interpolymer interaction is very strong; the polymers can block the pores of the ss-CaCO_3_ during multilayer deposition. One can then observe a predominant localization of the polymers on the edges of the ALG-based ss-MGs. For HS-based ss-MGs, the distribution of polymers is more homogeneous compared to ALG-based ones, which may be caused by the additional heating step during their preparation to improve the adhesion characteristics. The heating can promote polymer dynamics and make polymer distribution more homogeneous. The polymer distribution in the ss-MGs will be considered in our future works, where the analysis of the polymer distribution in ss-MGs can be performed as a function of molecular mass of encapsulated molecules and other related methods of analysis.

**Figure 8:**
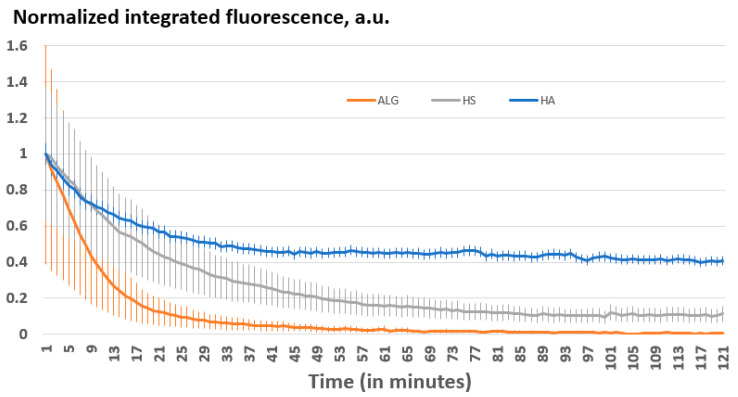
Normalized integrated fluorescence plotted against degradation time for bare ss-MGs.

As the next step, the kinetics of biodegradation have been examined based on the assessment of the integrated fluorescence from the ss-MGs (Figure 8). Among the different MGs, the degradation process was observed to be slowest in the HA-based ss-MGs, with approximately 60% of the ss-MGs undergoing degradation within a span of 1 h. Conversely, ALG-based ss-MGs exhibited the fastest degradation rate, characterized by the self-quenching effect described above that led to a surge in the background fluorescence at around the 30 min mark. The intensive release of PLL_FITC_ molecules may also result in higher fluorescent signals, which were observed for ALG-based ss-MGs (Figure 6). HS-based ss-MGs experienced substantial degradation, with approximately 90% degradation occurring within an hour. 

We assume that the ss-MGs with higher polymer dynamics (HA-based ones) possess slower degradation that may be related to the fact that they shrink more and form more compact multilayer structures with fewer defects (or water-filled voids) that restrict the diffusion of trypsin in the ss-MGs polymer network. In addition, for the HA-based ss-MGs, the degradation is not complete after 2 h, which may be related to the same explanation of a hindered diffusion of trypsin into the compact annealed structure of the multilayer complex, as discussed above. In contrast, for ALG- and HS-based ss-MGs, the degradation is faster and almost complete after 1–2 h, which can be explained by better accessibility of trypsin to the porous polymer network of the multilayers into these ss-MGs. At the same time, the additional crosslinking of Ca^2+^ ions into the ALG-based ss-MGs can be responsible for faster degradation as these are additional contacts present in only this type of ss-MGs analyzed in this work. A deeper analysis can be performed in a separate study to reveal the effect of calcium ions on ALG-based ss-MG properties.

Interestingly, the presence of DEX-FITC encapsulated into the ss-MGs suppresses the biodegradation (Figure S8) that may be caused by the slow diffusion of large 2000 kDa DEX-FITC molecules trapped into the multilayer network, which should be completely degraded to release all DEX-FITC content. We have chosen uncharged DEX-FITC as a model drug to avoid the interaction of the drug with biopolymers and, therefore, focus on the contribution of only interpolymer interactions in the biodegradation process. 

**Figure 9:**
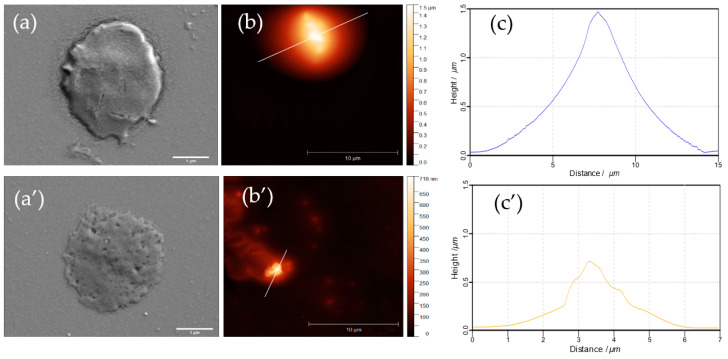
SEM (**a**,**a’**) and AFM (**b**,**b’**) images along with their height profile (**c**,**c’**) of HA-based ss-MGs before (**a**–**c**) and after 30 min biodegradation with trypsin (**a’**–**c’**). Scale bars for SEM and AFM images are 1 and 10 µm, respectively.

**Figure 10:**
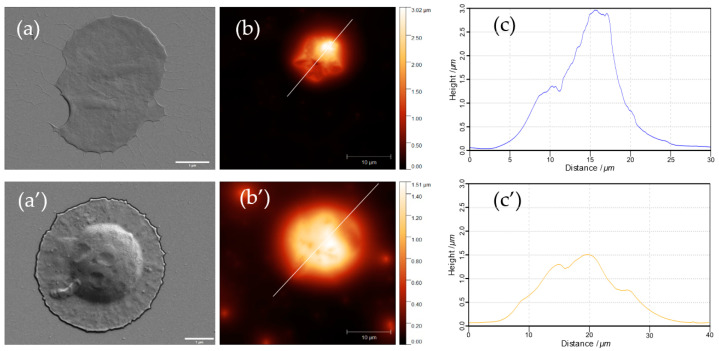
SEM (**a**,**a’**) and AFM (**b**,**b’**) micrographs along with their height profile (**c**,**c’**) of ALG-based ss-MGs before (**a**–**c**) and after 30 min biodegradation with trypsin (**a’**–**c’**). Scale bars for SEM and AFM images are 1 and 10 µm, respectively.

**Figure 11:**
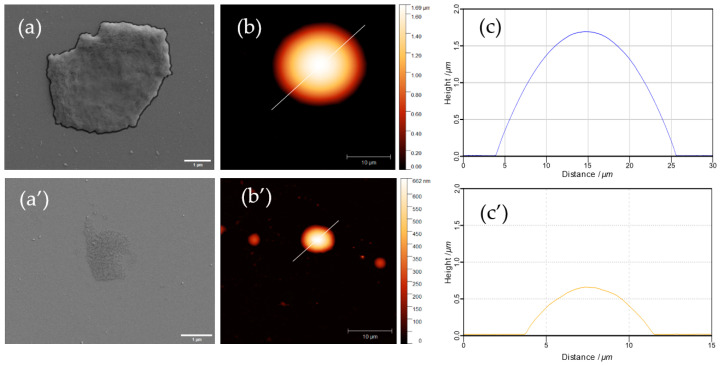
SEM (**a**,**a’**) and AFM (**b**,**b’**) micrographs along with their height profile (**c**,**c’**) of HS-based ss-MGs before (**a**–**c**) and after 30 min biodegradation with trypsin (**a’**–**c’**). Scale bars for SEM and AFM images are 1 and 10 µm, respectively.

To better understand the degradation process and the ss-MG structure, SEM and AFM have been employed, as discussed below (Figure 9, Figure 10 and Figure 11). It is of note that SEM and AFM images do not correspond to the same MGs as it is hard to analyze the same MGs using both techniques as they require sample preparation, including the formation of sample drying. The results of SEM and AFM demonstrate representative images of the ss-MGs before and after biodegradation in order to assess the ss-MG morphological changes. As evident from SEM images, after the biodegradation, a notable contrast in morphology for all ss-MGs is observed, featuring perforations on the external surface of the ss-MGs after degradation. Additionally, following degradation, a substantial reduction in height (AFM profiles) difference can be concluded. Interestingly, the morphology of HS-based ss-MGs is different compared to that of HA- and ALG-based ones. HS/PLL ss-MGs have a very smooth surface, which may be associated with the polymer annealing mediated by the heating of the formed ss-MGs during their formation. 

Overall, the results in the section above demonstrate that polymer interactions, i.e.**,** polymer chain dynamics, can play a main role in enzymatic degradation, as the dynamics define the process of annealing of multilayers. This, in turn, affects the structure of the multilayers and the diffusion of the enzyme molecules through the multilayers in order to reach the internal part of the MGs. For annealed multilayers, the enzymatic degradation rate is lower since pores/voids in the multilayers are closed, which hinders trypsin diffusion and further degradation. It is of note that the concentration of trypsin used in this study is comparable with that typically used for biodegradation in vitro experiments [46]. One should also note that other factors, such as pH and ionic strength, can significantly affect the enzymatic performance, and if pH and ionic strength are different inside multilayers compared to bulk solution, this can affect the biodegradation rate as well. This is, however, only a speculation and belongs to research questions of future work that can shed light on the effect of multilayer internal microenvironment on MG biodegradation. The trypsin solution used in this study has a neutral pH that can be an appropriate medium for the simulation of degradation of ss-MGs at the surface of implants where a pH near neutral is expected. For future works, it would be of interest to use alternative pH values to simulate other conditions relevant to drug delivery, e.g., low pH for oral delivery or alkaline pH for delivery in the gastrointestinal tract. 

#### 3.4.3. Formulation of Giant ss-MGs Using a One-Step ss-CaCO_3_ Synthesis 

**Figure 12:**
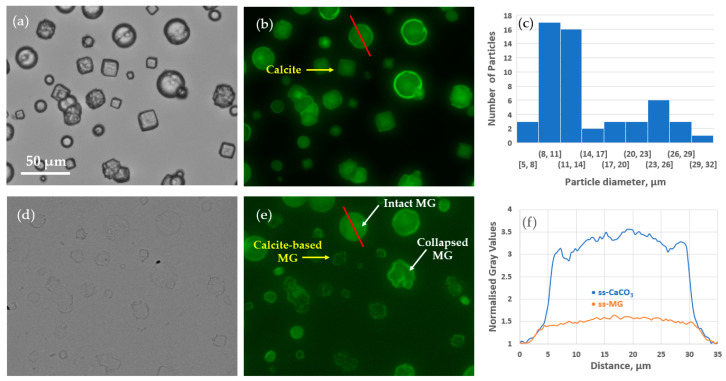
Optical microscopy transmittance and fluorescence image of ss-CaCO_3_ (**a**,**b**) and DEX-FITC loaded ss-HA/PLL ss-MGs (**d**,**e**), respectively. (**c**)—size distribution of the ss-CaCO_3_ crystals; horizontal axes show a range of sizes, i.e., from 8 to 11. (**f**)—normalized fluorescence profiles taken across particles in (**b**,**e**); red line shows the place where the profiles have been taken. The optical microscopy images are brightness and contrast-enhanced, so the profile shape can only be compared in (**f**). Yellow and white arrows point to the calcite-based ss-CaCO_3_ and ss-MGs and collapsed vaterite templated ss-MG, respectively. Of note, the ss-CaCO_3_ crystals fabricated using one-step approach; for details, see experimental section.

In the final step of this work, it is demonstrated that ss-CaCO_3_ can be fabricated in a one-step procedure via direct mixing of two precursor salts at shaking (for more details, see the experimental section). This allows the formulation of large vaterite crystals bound to the surface (Figure 12a–c). A typical batch of the crystals had a bimodal size distribution with well-defined particle populations of ca 10 and 23 µm in diameter (Figure 12c). The ss-MGs produced based on these crystals are also very large and can keep DEX-FITC encapsulated via pre-loading in preformed crystals (co-synthesis). Interestingly, the DEX-FITC molecules are distributed rather homogeneously in both ss-CaCO_3_ and ss-MGs (Figure 12f). Some ss-MGs can collapse, forming folds, but some keep their shape spherical (Figure 12b,e, white arrow). As soon as large ss-CaCO_3_ is synthesized, calcite accompanies the vaterite. Cubic-shaped calcite crystals (yellow arrow in Figure 12) can host less DEX-FITC as they have a non-porous structure compared to mesoporous vaterite. However, stable ss-MGs can also be formed for calcite crystals. The focus of our ongoing research is to understand the structure and formation mechanism of ss-MGs along with ss-CaCO_3_.

## 4. Conclusions

The primary objective of this research is two-fold: first, to explore the feasibility of forming multilayer microgels onto the surface utilizing ss-CaCO_3_ as a sacrificial template, and second, to undercover the underlying mechanism of ss-MGs biodegradation for the purpose of finely tuning drug release profiles. This study aims to shed light on the intriguing potential of ss-MGs for localized drug delivery applications, wherein the enzyme-stimulated degradation of these microgels can be harnessed to precisely regulate the release of therapeutic agents. 

Our study has provided comprehensive insights into the various facets of microgel behavior involving formation, adhesion, shrinking, stability, and controlled drug release mechanisms. A clear link has been established between the formation yield and subsequent adhesion in ALG, HA, and HS-based ss-MGs. Shrinkage of MGs correlates with biopolymer composition—ALG microgels exhibit minimal shrinking due to rigid structure and strong interpolymer interactions, showing no polymer annealing and porous polymer multilayer network, while HA-based ss-MGs show pronounced shrinking from the annealing process. The annealing of HS-based ss-MGs can only be mediated via the heating of the formed MGs; otherwise, they do not stick to the surface. Dextran-loading improves the formation yield and reduces shrinking tendencies, suggesting the potential for customized microgel design. Additionally, we uncovered a connection between polymer chain dynamics and ss-MG biodegradation, implying slower degradation rates with higher polymer chain dynamics and constrained trypsin diffusion. Finally, it is demonstrated that the fabrication of ss-MGs can be possible using a one-step simple formation of CaCO_3_ followed by a multilayer coating of surfaces via simple replacing of polymer and rinsing solutions in cell culture plate wells. This can offer a way to test such ss-MGs for cell culture experiments in wells, which is very convenient. 

By understanding both the formation process of ss-MGs on the surface and the intricate degradation pathways they undergo, we aspire to pave the way for the development of innovative drug delivery systems with enhanced precision and efficacy. These findings hold significant implications for advancing drug delivery strategies and expanding the frontiers of biomedical research. Alternative applications of the ss-MGs can be found in other fields, such as analytics and drug testing. The ss-MGs can serve as small sensors with integrated analytical moieties (e.g., as constituents of multilayers) to determine analytes in bulk; easy-to-separate ss-MGs from the bulk will be a significant advantage. Bio-applications will still remain a primary focus as the proposed technology offers a fully biocompatible process for the immobilization of fragile bioactives (e.g., growth factors and hormones) and controlled localization of the bioactives on a surface, which also offers a way to regulate cellular adhesion as a multilayer is a perfect platform for cell culture studies. It is currently a challenge to design the interface between living tissue and a scaffold/implant, which is key for proper bio-integration.

## Figures and Tables

**Table 1:** Average diameter (µm) of the ss-CaCO_3_ and MGs templated on them.

	HA/PLL MGs	ALG/PLL MGs	HS/PLL MGs
ss-CaCO_3_	18.1 ± 1.3	16.9 ± 0.8	14.6 ± 0.7
ss-MGs	5.5 ± 1.0	15.4 ± 0.8	7.8 ± 1.6
DEX-FITC loaded ss-CaCO_3_	9.3 ± 0.7	8.3 ± 0.7	9.9 ± 0.6
DEX-FITC loaded ss-MGs	7.3 ± 0.6	9.9 ± 0.6	9.2 ± 0.6

## Data Availability

The data presented in this study are available on request from the corresponding author.

## Supplementary Materials

The following supporting information can be downloaded at https://www.mdpi.com/article/10.3390/ma17010103/s1, Figure S1: Transmittance and fluorescence images of ss-CaCO_3_ synthesized before (a) washing and after (b) washing with 0.2x Tris buffer; Figure S2: Transmittance images of ss-CaCO_3_ crystals synthesized (a) and of HA/PLL fabricated ss-MGs (b). Scale bar is 200 μm; Figure S3: Transmittance images of ss-CaCO_3_ crystals synthesized (a) and of HS/PLL fabricated ss-MGs (b). Scale bar is 200 μm; Figure S4: Transmittance and fluorescence microscopy images of HS/PLL coated ss-CaCO_3_ (a) and corresponding ss-MG (b). ss-MG after washing with 0.2x TRIS buffer. Edited brightness and contrast; Figure S5: Fluorescence profiles of (HA/PLL)2/HA coated ss-CaCO_3_ before (a) and after (d) overnight freezing. Corresponding optical microscopy transmittance (b,c) and fluorescence (e,f) images before (b,e) and after (c,f) overnight freezing; Figure S6: Fluorescence profiles of (ALG/PLL)2/ALG coated ss-CaCO_3_ before (a) and after (d) overnight freezing. Corresponding optical microscopy transmittance (b,c) and fluorescence (e,f) images before (b,e) and after (c,f) overnight freezing; Figure S7: Fluorescence profiles of (HS/PLL)2/HS coated ss-CaCO_3_ before (a) and after (d) overnight freezing. Corresponding optical microscopy transmittance (b,c) and fluorescence (e,f) images before (b,e) and after (c,f) overnight freezing; Figure S8: Normalized integrated fluorescence plotted against degradation time DEX-FITC loaded MGs.
