# Supplementary material for: Formulation and Biodegradation of Surface-Supported Biopolymer-Based Microgels Formed via Hard Templating onto Vaterite CaCO3 Crystals
Source: Materials (Basel). 2023 Dec 25;17(1):103. doi: 10.3390/ma17010103 (PMC10779910; doi:10.3390/ma17010103)
Supplement: Supplementary file 1 [file materials-17-00103-s001.zip › materials-2764782-supplementary.pdf]

SUPPORTING INFORMATION

Formulation and biodegradation of surface-supported biopolymer microgels formed via hard templating onto vaterite CaCO3 crystals

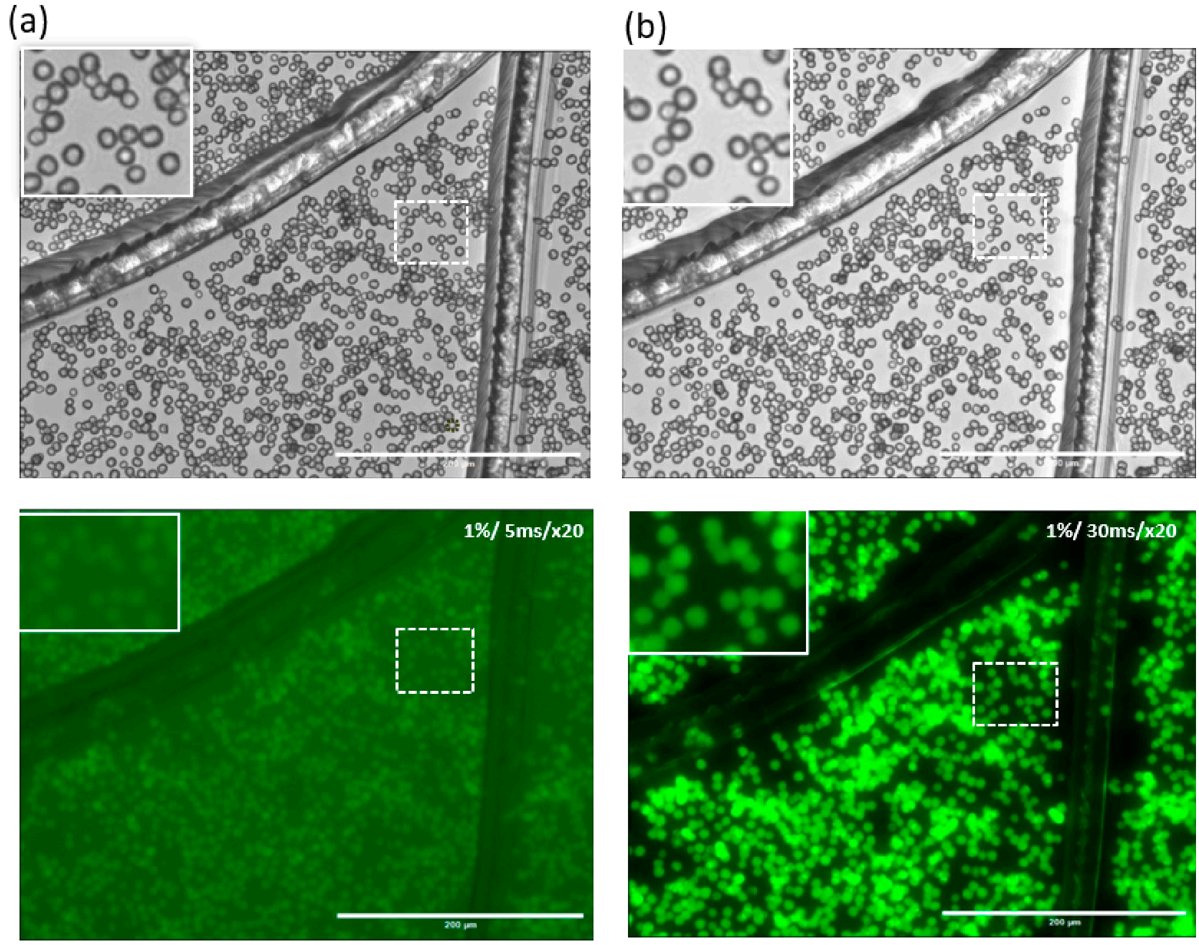

Figure S1. Transmittance and fluorescence images of ss-$CaCO_3$ synthesized before (a) washing and after (b) washing with 0.2x Tris buffer. Scale bar is 200 μm.

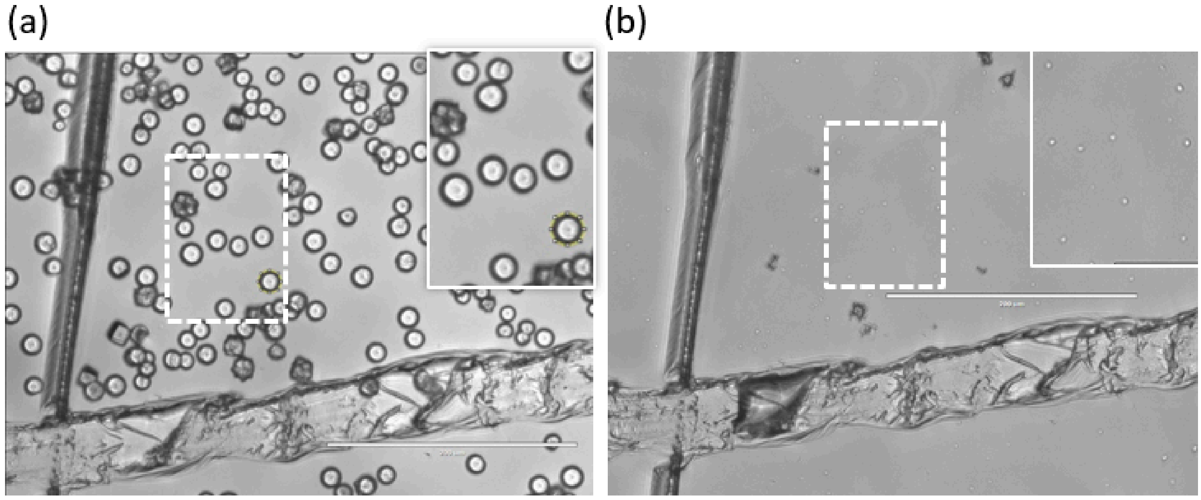

Figure S2. Transmittance images of ss-$CaCO_3$ crystals synthesized (a) and of HA/PLL fabricated ss-MGs (b). Scale bar is 200 μm.

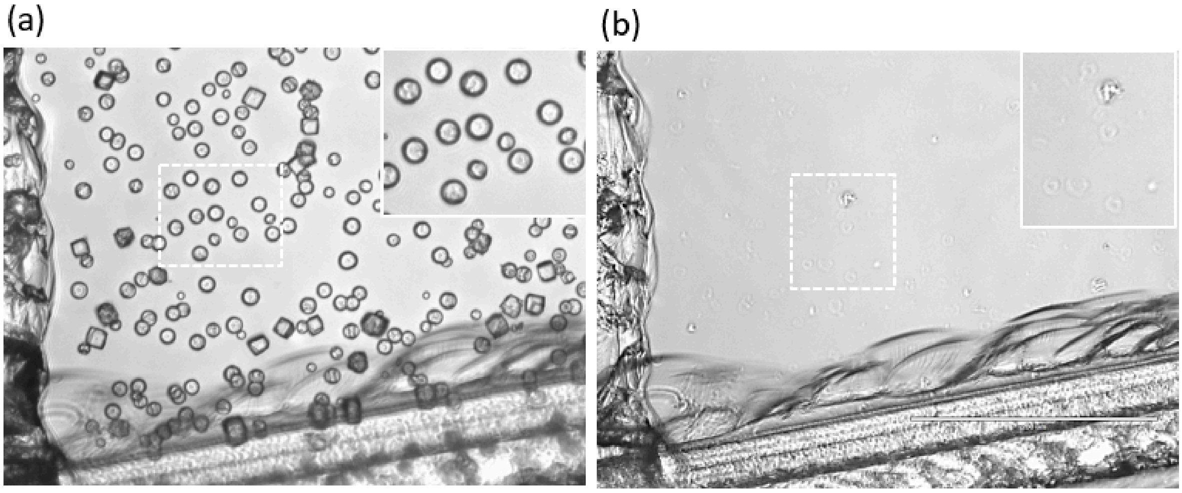

Figure S3. Transmittance images of ss-$CaCO_3$ crystals synthesized (a) and of HS/PLL fabricated ss-MGs (b). Scale bar is 200 μm.

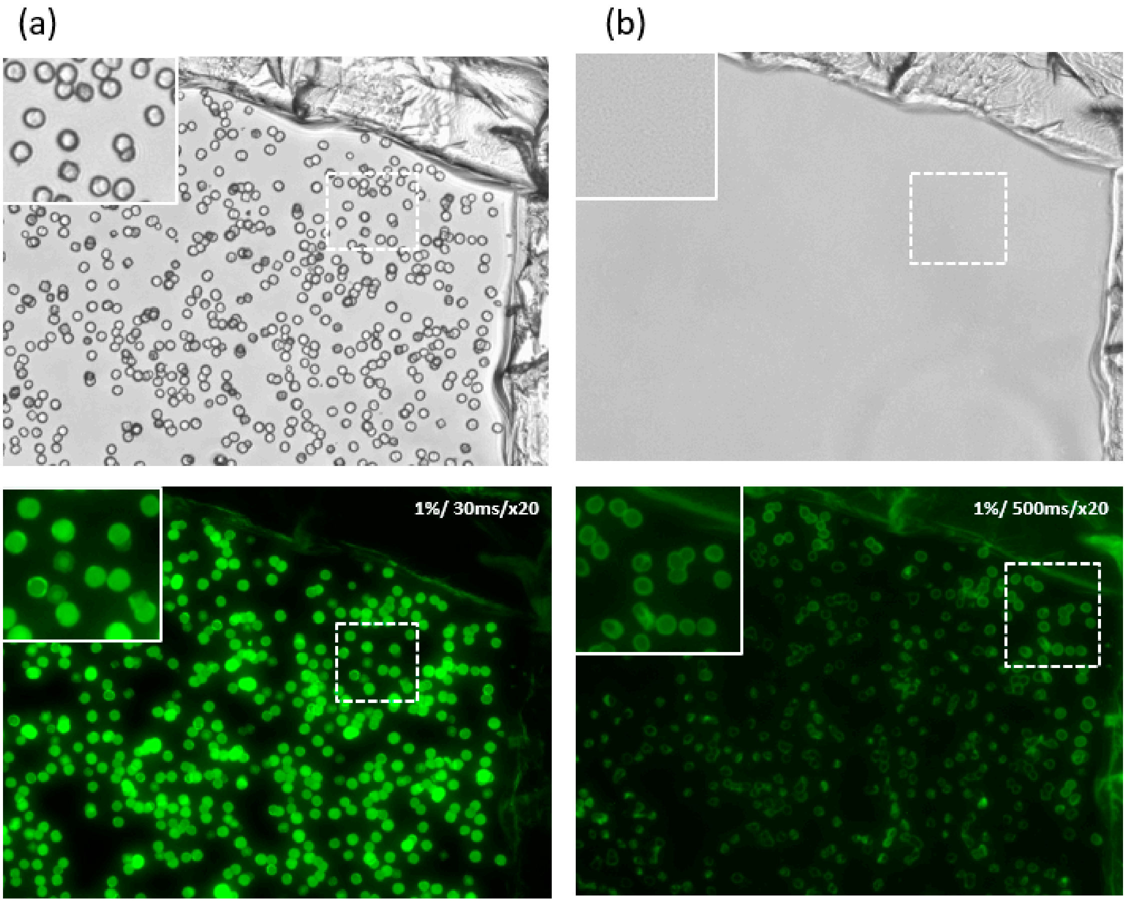

Figure S4. Transmittance and fluorescence microscopy images of HS/PLL coated ss-$CaCO_3$ (a) and corresponding ss-MG (b). ss-MG after washing with 0.2x TRIS buffer. Edited brightness and contrast.

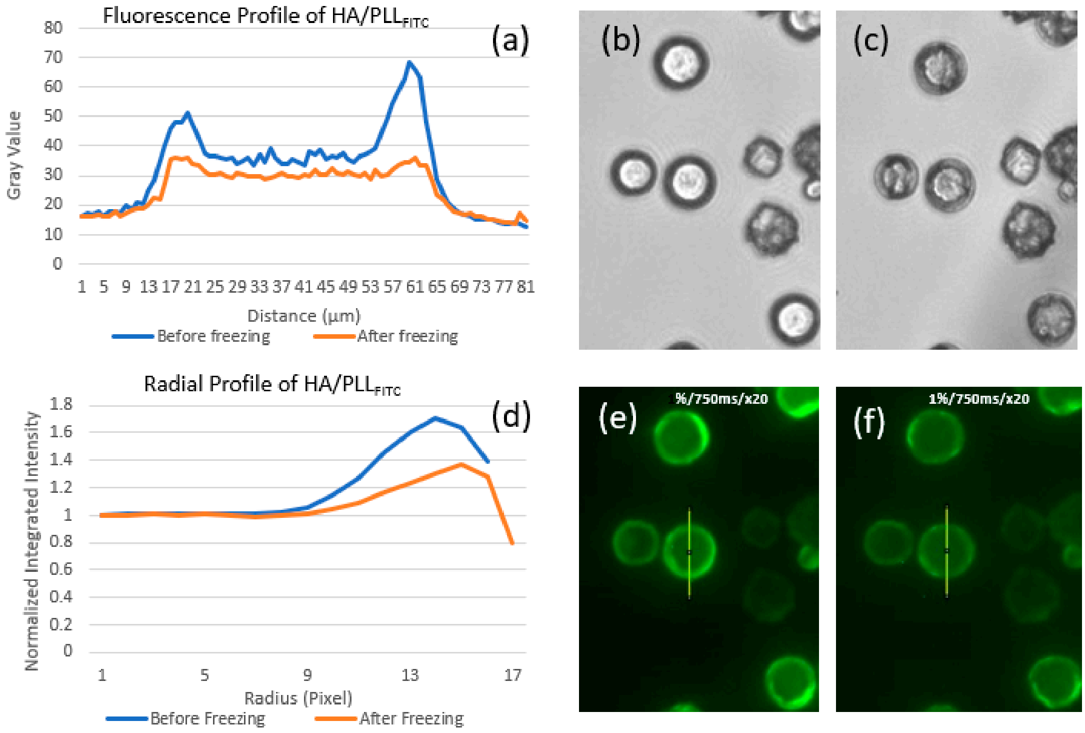

Figure S5. Fluorescence profiles of (HA/PLL)$_2$/HA coated ss-$CaCO_3$ before (a) and after (d) overnight freezing. Corresponding optical microscopy transmittance (b,c) and fluorescence (e,f) images before (b,e) and after (c,f) overnight freezing.

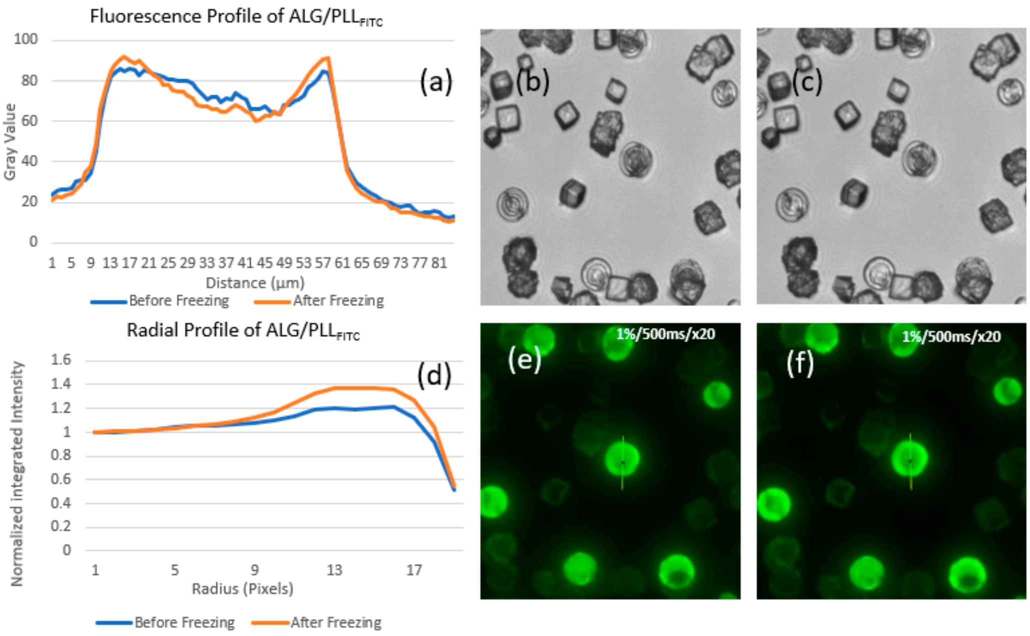

Figure S6. Fluorescence profiles of (ALG/PLL)$_2$/ALG coated ss-$CaCO_3$ before (a) and after (d) overnight freezing. Corresponding optical microscopy transmittance (b,c) and fluorescence (e,f) images before (b,e) and after (c,f) overnight freezing.

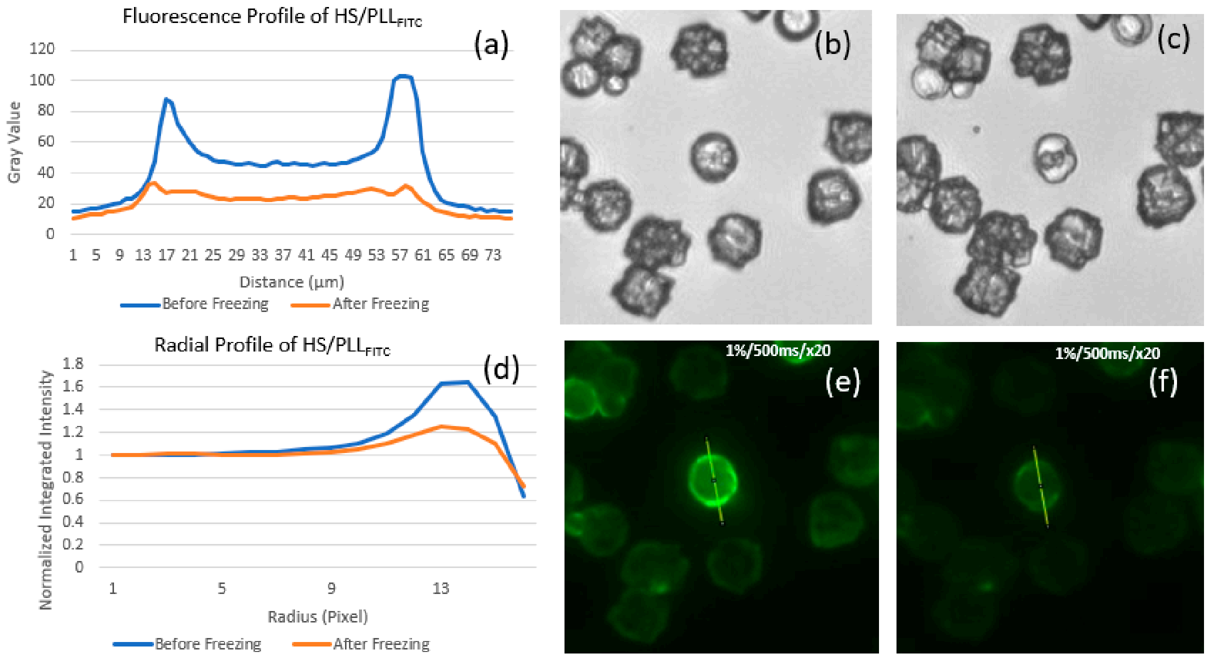

Figure S7. Fluorescence profiles of $(HS/PLL)_2/HS$ coated ss-$CaCO_3$ before (a) and after (d) overnight freezing. Corresponding optical microscopy transmittance (b,c) and fluorescence (e,f) images before (b,e) and after (c,f) overnight freezing.

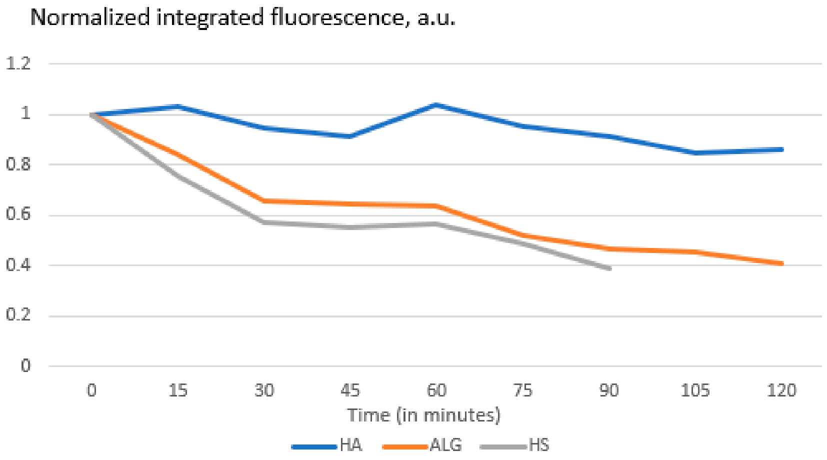

Figure S8. Normalized integrated fluorescence plotted against degradation time DEX-FITC loaded MGs.
